# Scattering on a square lattice from a crack with a damage zone

**DOI:** 10.1098/rspa.2019.0686

**Published:** 2020-03-18

**Authors:** Basant Lal Sharma, Gennady Mishuris

**Affiliations:** 1 Department of Mechanical Engineering, Indian Institute of Technology Kanpur, Kanpur, India; 2 Department of Mathematics, Aberystwyth University, Aberystwyth, UK

**Keywords:** diffraction, crack, damage zone, Wiener–Hopf method

## Abstract

A semi-infinite crack in an infinite square lattice is subjected to a wave coming from infinity, thereby leading to its scattering by the crack surfaces. A partially damaged zone ahead of the crack tip is modelled by an arbitrarily distributed stiffness of the damaged links. While an open crack, with an atomically sharp crack tip, in the lattice has been solved in closed form with the help of the scalar Wiener–Hopf formulation (Sharma 2015 *SIAM J. Appl. Math.*, **75**, 1171–1192 (doi:10.1137/140985093); Sharma 2015 *SIAM J. Appl. Math.* **75**, 1915–1940. (doi:10.1137/15M1010646)), the problem considered here becomes very intricate depending on the nature of the damaged links. For instance, in the case of a partially bridged finite zone it involves a 2 × 2 matrix kernel of formidable class. But using an original technique, the problem, including the general case of arbitrarily damaged links, is reduced to a scalar one with the exception that it involves solving an auxiliary linear system of *N* × *N* equations, where *N* defines the length of the damage zone. The proposed method does allow, effectively, the construction of an exact solution. Numerical examples and the asymptotic approximation of the scattered field far away from the crack tip are also presented.

## 1. Introduction

Among other distinguished as well as popular works [1], Peter Chadwick made several contributions to the wave propagation problems in anisotropic models with different kinds of symmetries as well as those applicable to the theory of lattice defects [2–8]. His research into elastic cubic crystals is especially relevant in the context of the present paper as a discrete counterpart of a square lattice is natural when one considers waves interacting with a crack tip [9–14].

Indeed, the role of discrete models in the description of the mechanics and physics of crystals [15] and related structures has dominated studies of several critical phenomena, such as dislocation dynamics, dynamic fracture and phase transition, bridge crack effects, and resonant primitive, localized and dissipative waves in lattices among others [16–32]. The concomitant issues dealing with the propagation of waves interacting with stationary cracks and rigid constraints as well as surface defects have been explored in [12–14,33–42]. It is noteworthy that the continuum limit, which is a low-frequency approximation of the scattering problem for a single crack [13,43], recovers the well-known solution of Sommerfeld [44,45]. With respect to the crack-tip geometry, note that the discrete scattering problems have been solved in [12,13,35,38,40] for atomically sharp crack tips.

**Figure 1.**
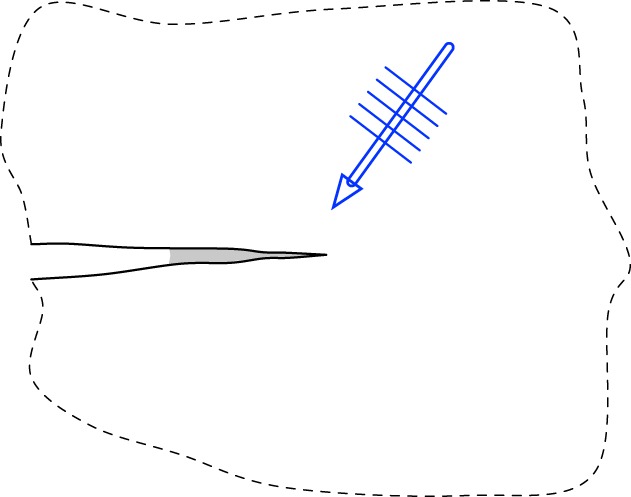
Schematic of an incident wave on a crack tip with damage. (Online version in colour.)

Typically, such situations of discrete scattering due to crack surfaces are further complicated as the crack tip is endowed with some structure, as shown schematically in figure 1, due to the presence of a cohesive zone, partial bridging of bonds, etc., commonly used in continuum mechanics [46–48]. The notion of a cohesive zone used in this paper is considered in a wider sense than in fracture mechanics (it does not clearly eliminate any singularities that do not arise in the discrete formulation). The zone simply emphasizes the fact that different links, subjected to a high-amplitude vibration, near the crack tip may undergo phase transition, damage and/or breakage at different times depending on the material’s properties (manifesting by respective damage/fracture criteria [49]). As a result, a naturally created partial bridging and/or forerunning zone can be observed during crack propagation (e.g. [30,50]).

The problem considered in this paper, in fact, becomes much more intractable when compared with the scattering due to an atomically sharp crack tip that has been solved in [12,13] using the scalar Wiener–Hopf factorization [11,51]. As an example, it is shown that, in the case of a partially bridged finite zone, the corresponding Wiener–Hopf problem becomes vectorial as it involves a 2 × 2 matrix kernel that belongs to a formidable class [52–55]. In this paper, it is shown that a reduction to a scalar problem is possible with the additional clause that it involves solving an auxiliary linear system of *N* × *N* equations, where *N* represents the size of the cohesive zone. Such a reduction resembles the one proposed for the Wiener–Hopf kernel with exponential phase factors in the continuum case [52–55], and its recently investigated discrete analogue of scattering due to a pair of staggered crack tips [34,56]. It is also relevant to recall for such kernels an asymptotic factorization-based alternative, but approximate, approach [34,57].

Overall, the method proposed in this paper does allow, effectively, the construction of an exact solution, even in the general case of an arbitrary set of damaged links. The paper presents some numerical examples to demonstrate the effect of certain kinds of damaged links on the pattern of a scattered field. The expression obtained after an asymptotic approximation of the scattered field far away from the crack tip is also presented as a perturbation over and above that for the atomically sharp crack tip obtained earlier in [12]. A careful analysis of the continuum limit [43], in the presence of damaged links, which demands adoption of a proper scaling, is relegated to future work. The question of the behaviour of edge conditions with regard to sharp cracks [51,58] is anticipated to be crucial in such an exercise.

As a summary of the organization and presentation of the main aspects of this paper, §2 gives the mathematical formulation of the scattering problem. Section 3 provides the exact solution of the Wiener–Hopf equation modulo the reduced form to an auxiliary linear system of *N* × *N* equations. Section 4 presents some special scenarios of the distribution of the damaged links that allow either an immediate solution of the auxiliary equation or demonstrate the difficulty and richness of the problem by mapping its difficulty to a class of problems. Section 5 gives the far-field behaviour away from the crack tip as a perturbation in addition to that for a sharp crack tip, as well as some numerical examples. Section 6 concludes the findings of this paper. Appendix A gives the technical details of the application of the Wiener–Hopf method. For details of the theory of scattering and the Wiener–Hopf method, we refer to [51,59]; the mathematical aspects of convolution integrals and Fourier analysis can be found in [60–66]. For the issues dealing with the difficult cases of the matrix Wiener–Hopf problems, the reader is referred to [57,67–73].

## 2. Problem formulation

Let us consider a square lattice structure consisting of a semi-infinite crack that involves an additional structural feature near the crack tip. The bulk lattice is constructed with the same masses, *m*, situated at the points (*x*, *y*), x∈Z, y∈Z and connected by elastic springs with stiffness, *c* > 0 (figure 1). The space coordinates are dimensionless and define the position of the corresponding mass $\left(x,y)=(\tilde{x}/a,\tilde{y}/a\right)$ (normalized by the length of the links between the neighbouring masses *a*). Displacement of the mass at each point is denoted as *u*_*x*,*y*_(*t*).

**Figure 2.**
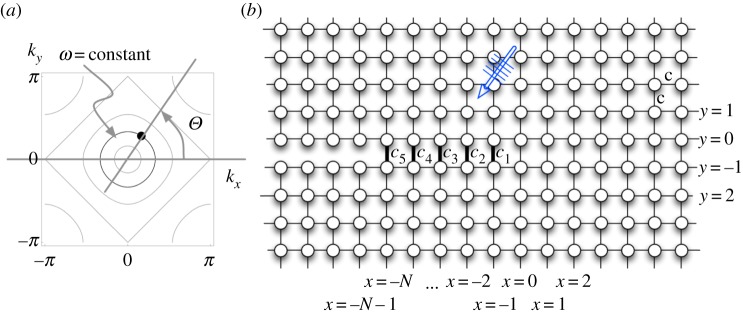
(*a*) Schematic of the incident wave parameters relative to the typical contours for a square lattice dispersion relation. (*b*) Geometry of the square lattice structure and the notation for the number of damaged sites *N* = 5. (Online version in colour.)

The bonded interface between the two half-planes consists of a finite segment of distributed springs of stiffness, ${\left\{c_{-x}\right\}}_{x=-1}^{-N}$ (*c*_−*x*_ ≥ 0), with connecting masses from the different sides of the interface attributed to the values of the variable *x* (figure 2). Note that some of the links can also be considered fully destroyed; thus, the geometry of the damage zone can be rather complex.

In the following, we will use the standard notation

2.1 $$Z^{+}=\{0,1,2,\ldots \},\;Z^{-}=\{-1,-2,\ldots \}\;\text{and}\;Z=Z^{+}\cup Z^{-}.$$

We assume that an incident wave

2.2 $$u^{i}(x,y,t)=u_{x,y}^{i}{\text{e}}^{-i\omega t}=A{\text{e}}^{-ik_{x}x-ik_{y}y-i\omega t}$$

imposes the out-of-plane small deformation of the lattice. Here, $k_{x},k_{y}\in R$ are wavenumbers; also, sometimes we use *k*_*x*_ = *k*cosΘ, *k*_*y*_ = *k*sinΘ with *k* > 0 and Θ ∈ [ − *π*, *π*]. The symbol A∈C is the complex dimensional amplitude of the wave. It is further assumed that *ω* = *ω*_1_ + *iω*_2_ (where *ω*_2_ > 0 is an arbitrary small number). The latter guarantees that the causality principle is addressed. Note that this implies *k* = *k*_1_ + *ik*_2_, where *k*_2_ is small when *ω*_2_ is small. We seek the harmonic solution to the problem of the form

2.3 $$u^{t}(x,y,t)=u_{x,y}^{t}{\text{e}}^{-i\omega t}=(u_{x,y}^{s}+u_{x,y}^{i}){\text{e}}^{-i\omega t},$$

where $u_{x,y}^{s}$ and $u_{x,y}^{i}$ are the scattered part and the incident part, respectively.

The following set of equations are valid in each part of the lattice structure outside the interphase (*y* ≥ 1 and *y* ≤ −2):

2.4 $$c\Delta u_{x,y}+\omega^{2}u_{x,y}=0,\;x\in Z.$$

Here, Δ is the discrete Laplace operator with Δ*u*_*x*,*y*_ = *u*_*x*+1,*y*_ + *u*_*x*−1,*y*_ + *u*_*x*,*y*+1_ + *u*_*x*,*y*−1_ − 4*u*_*x*,*y*_ (see [11,12,74]) and in the following $u_{x,t}=u_{x,y}^{t}$.

The interphase consists of two lines *y* = 0 and *y* = −1 (figure 1). Let the damaged portion be denoted by the values of the coordinate *x* lying in

2.5 D={−1,−2,…,−N}.

Let us denote the Kronecker delta by the symbol *δ*; it is equal to unity when x∈D and zero otherwise. Also we denote the discrete Heaviside function by *H*(*x*) for x∈Z, defined such that *H*(*x*) = 1 if $x\in Z_{+}$, while *H*(*x*) = 0 when $x\in Z_{-}$. Furthermore, let us introduce the notation

2.6 $$v_{x}=(u_{x,0}-u_{x,-1}),\;v_{x}^{i,s}=(u_{x,0}^{i,s}-u_{x,-1}^{i,s}),\;x\in Z.$$

As a result, for x∈Z, the conditions $c\Delta u_{x,0}+(c-c_{-x}\delta_{D,x}-cH(x))v_{x}+\omega^{2}u_{x,0}=0$ and $c\Delta u_{x,-1}-(c-c_{-x}\delta_{D,x}-cH(x))v_{x}+\omega^{2}u_{x,-1}=0$, linking the top part of the lattice with the bottom part, can be written as

2.7 $$c\Delta u_{x,0}^{s}+(c-c_{-x}\delta_{D,x}-cH(x))v_{x}^{s}+\omega^{2}u_{x,0}^{s}=-(c-c_{-x}\delta_{D,x}-cH(x))v_{x}^{i}$$

and

2.8 $$c\Delta u_{x,-1}^{s}-(c-c_{-x}\delta_{D,x}-cH(x))v_{x}^{s}+\omega^{2}u_{x,-1}^{s}=(c-c_{-x}\delta_{D,x}-cH(x))v_{x}^{i}.$$

The skew symmetry follows immediately, i.e.

2.9 $$u_{x,-1}^{s}+u_{x,0}^{s}=0,\;x\in Z,$$

and in general $u_{x,-y-1}^{s}+u_{x,y}^{s}=0$, $y\in Z^{+}$. Hence, it is enough to look at *y* = 0, or a difference of equations (2.7) and (2.8). Let A be an appropriate annulus in the complex plane, the same as that stated in [12], i.e.

2.10 $$A:=\{z\in C:R_{+}<|z|<R_{-}\},\;R_{+}={\text{e}}^{-k_{2}}\;\text{and}\;R_{-}={\text{e}}^{k_{2}\cos\Theta }.$$

Taking into account the skew symmetry of the problem under consideration (see [12] and (2.9)), we conclude that

2.11 $$v^{F}=2u_{0}^{F}.$$

Applying the Fourier transform

2.12 $$u^{F}(z)\equiv \sum_{x\in Z}z^{-x}u_{x}^{s},\;z\in A,$$

to equation (2.4) for scattering waves in the upper space *y* ≥ 0, we obtain, following Slepyan [11] and Sharma [12],

2.13 $$u_{y}^{F}(z)=\lambda^{y}(z)u_{0}^{F}(z),\;y=0,1,2,\ldots ,\;z\in A,$$

with

2.14 $$\lambda (z)=\frac{r(z)-h(z)}{r(z)+h(z)},\;h(z)=\sqrt{Q(z)-2},\;r(z)=\sqrt{Q(z)+2}$$

and

2.15 $$Q(z)=4-z-z^{-1}-\omega^{2}.$$

An analogous result can be obtained in the lower space (*y* ≤ −1). The details are identical to those for a crack without a damage zone as provided in [12].

Taking into account the condition (2.9) as well as (2.13) (in particular, $u_{1}^{F}=-u_{-2}^{F}=u_{0}^{F}\lambda$ with *λ* given by (2.14)), we obtain

2.16 $$c\left(\lambda -1-Q(z)\right)v^{F}(z)+2cv_{-}(z)-cP^{s}(z)=-2cv_{-}^{i}(z)+cP^{i}(z).$$

Thus, with *v*^*F*^ = *v*_+_ + *v*_−_, we have

2.17 $$(\lambda -Q-1)(v_{+}+v_{-})+2v_{-}=-2v_{-}^{i}+P^{i}+P^{s},$$

i.e.

2.18 $$v_{+}+\left(\frac{2}{\lambda -Q-1}+1\right)v_{-}=-\frac{2}{\lambda -Q-1}v_{-}^{i}+\frac{1}{\lambda -Q-1}(P^{i}+P^{s}).$$

Simplifying further for z∈A, we get

2.19 $$v_{+}+Lv_{-}=(1-L)v_{-}^{i}-\frac{1}{2}(1-L)\left(P^{i}(z)+P^{s}(z)\right),$$

where *L*(*z*) = *h*(*z*)/*r*(*z*), while $P^{s}$ (and $P^{i}$) is a polynomial in *z* given by

2.20 $$P^{s,i}(z)=\frac{2}{c}\sum_{x\in D}c_{-x}v_{x}^{s,i}z^{-x}.$$

Equation (2.19) is the Wiener–Hopf equation for the Fourier transform of the bonds *v*_±_ in the cracked row ($x\in Z^{\pm }$). Inspection and comparison with the results for a single crack without the damage zone obtained in [12] reveals that the kernel remains the same but there is a presence of an extra unknown polynomial on the right-hand side of the Wiener–Hopf equation.

## 3. Solution of the Wiener–Hopf equation

The relevant multiplicative factorization of the kernel *L* in (2.19) on the annulus A, i.e. *L* = *L*_+_*L*_−_, has been obtained in an explicit form in equation (2.27) from [12]. Thus, using this fact, (2.19) can be written as

3.1 $$L_{+}^{-1}v_{+}+L_{-}v_{-}=C\;\text{on}\;A,$$

where $C=C^{a}+C^{P^{s}}$ and

3.2 $$\begin{array}{rl} & C^{a}(z)=(L_{+}^{-1}(z)-L_{-}(z))v_{-}^{i}(z)\\ \text{and}\; & C^{P}(z)=-\frac{1}{2}(L_{+}^{-1}(z)-L_{-}(z))\left(P^{i}(z)+P^{s}(z)\right). \end{array}\}$$

Note that *L*_+_ is analytic and non-vanishing for |*z*| > *R*_+_ and that *L*_−_ is analytic and non-vanishing for |*z*| < *R*_−_. Now

3.3 $$v_{-}^{i}(z)=A(1-{\text{e}}^{ik_{y}})\delta_{D-}(zz_{P}^{-1}),\;z_{P}={\text{e}}^{-ik_{x}}$$

(note that $|z_{P}|>R_{-}$ in (2.10)), so that

3.4 $$C^{a}=C_{+}^{a}+C_{-}^{a},$$

with [12]

3.5 $$C_{+}^{a}=\left(L_{+}^{-1}-L_{+}^{-1}(z_{P})\right)v_{-}^{i}\;\text{and}\;C_{-}^{a}=\left(-L_{-}+L_{+}^{-1}(z_{P})\right)v_{-}^{i}.$$

Here, $C_{+}^{a}$ is analytic for |*z*| > *R*_+_ and $C_{-}^{a}$ is analytic for |*z*| < *R*_−_. Let $P^{t}(z)$ denote the sum $P^{s}(z)+P^{i}(z)$ (with the coefficients $v_{x}^{t}=v_{x}^{s}+v_{x}^{i}$), i.e.

3.6 $$P^{t}(z)=\frac{2}{c}\sum_{x\in D}c_{-x}v_{x}^{t}z^{-x}.$$

Further (recall that D is defined in (2.5))

3.7 $$L_{+}^{-1}(z)P^{t}(z)=\frac{2}{c}\sum_{x\in D}c_{-x}v_{x}^{t}(L_{+}^{-1}(z)z^{-x}),\;z\in A.$$

Using the expressions from [13], $L_{+}^{-1}$ can be expanded in a series of the form

$$L_{+}^{-1}(z)=\sum_{m\in Z^{+}}{\bar{l}}_{+m}z^{-m},$$

for |*z*| > *R*_+_. Thus (with $x=-\nu \in Z^{-}$)

3.8 $$L_{+}^{-1}(z)z^{-x}=\sum_{m\in Z^{+}}{\bar{l}}_{+m}z^{-m}z^{\nu }=\phi_{+}^{x}(z)+\phi_{-}^{x}(z),\;z\in A,$$

where

3.9 $$\phi_{+}^{x}(z)=\sum_{m=\nu }^{\infty }{\bar{l}}_{+m}z^{-m}z^{\nu }\;\text{and}\;\phi_{-}^{x}(z)=\sum_{m=0}^{\nu -1}{\bar{l}}_{+m}z^{-m}z^{\nu },$$

and the first term is analytic outside a circle of radius *R*_+_ while the second is analytic inside a circle of radius *R*_−_ in the complex plane. Therefore, in the context of (3.7),

3.10 $$L_{+}^{-1}P^{t}=\frac{2}{c}\sum_{x\in D}c_{-x}v_{x}^{t}\phi_{+}^{x}+\frac{2}{c}\sum_{x\in D}c_{-x}v_{x}^{t}\phi_{-}^{x}.$$

The above additive splitting of $L_{+}^{-1}P^{t}$, naturally, allows the following additive decomposition:

3.11 $$C^{P}=C_{+}^{P}+C_{-}^{P}\;\text{on}\;A,$$

where

3.12 $$C_{+}^{P}=-\frac{1}{c}\sum_{x\in D}c_{-x}v_{x}^{t}\phi_{+}^{x}\;\text{and}\;C_{-}^{P}=-\frac{1}{c}\sum_{x\in D}c_{-x}v_{x}^{t}\phi_{-}^{x}+\frac{1}{2}L_{-}P^{t},$$

which are analytic outside and inside of a circle of radius *R*_+_ and *R*_−_ in the complex plane, respectively. As a final step, following the analysis in [12] and using the expressions (3.4), (3.11) and (2.19) leads to

3.13 $$\begin{array}{rl} & L_{+}^{-1}(z)v_{+}(z)=C_{+}^{a}(z)+C_{+}^{P}(z)+\chi (z),\;|z|>R_{+},\\ \;\text{and}\; & L_{-}(z)v_{-}(z)=C_{-}^{a}(z)+C_{-}^{P}(z)-\chi (z),\;|z|<R_{-}, \end{array}\}$$

where *χ* is an arbitrary polynomial in *z* and *z*^−1^. It is shown in [12] that, as *z* → ∞,

$$L_{+}^{-1}v_{+}-C_{+}^{a}-C_{+}^{P}\to \text{constant},$$

while as *z* → 0

$$L_{-}v_{-}-C_{-}^{a}-C_{-}^{P}\to 0,$$

so that, as a consequence of Liouville’s theorem, *χ* ≡ 0. Hence,

3.14 $$\begin{array}{rl} & v_{+}(z)=L_{+}(z)(C_{+}^{a}(z)+C_{+}^{P}(z)),\;|z|>R_{+},\\ \;\text{and}\; & v_{-}(z)=L_{-}^{-1}(z)(C_{-}^{a}(z)+C_{-}^{P}(z)),\;|z|<R_{-}. \end{array}\}$$

Owing to (3.14)

3.15 $$\begin{array}{rl} v^{F}(z) & =L_{+}(z)(C_{+}^{a}(z)+C_{+}^{P}(z))+L_{-}^{-1}(z)(C_{-}^{a}(z)+C_{-}^{P}(z))\\ & =v_{a}^{F}(z)+v_{P}^{F}(z),\;z\in A, \end{array}$$

with

3.16 $$v_{a}^{F}=L_{+}C_{+}^{a}+L_{-}^{-1}C_{-}^{a}\;\text{and}\;v_{P}^{F}=L_{+}C_{+}^{P}+L_{-}^{-1}C_{-}^{P}.$$

Also the total field *v* (the total oscillatory field along the symmetry axis) is given by

3.17 $$v_{x}=v_{x}^{i}+v_{x}^{s}=v_{x}^{i}+\frac{1}{2\pi i}\int_{C}v^{F}(z)z^{x-1}\;\text{d}z,\;x\in Z.$$

In particular, expanding (3.14)_2_ further,

3.18 $$v_{-}=(-1+L_{-}^{-1}L_{+}^{-1}(z_{P}))v_{-}^{i}-\frac{1}{c}L_{-}^{-1}\sum_{x\in D}c_{-x}v_{x}^{t}\phi_{-}^{x}+\frac{1}{2}P^{t},$$

with |*z*| < *R*_−_. Re-arranging (3.18), we get

3.19 $$v_{-}(z)+v_{-}^{i}(z)=L_{+}^{-1}(z_{P})\frac{v_{-}^{i}(z)}{L_{-}(z)}-\frac{1}{c}L_{-}^{-1}(z)\sum_{x\in D}c_{-x}v_{x}^{t}\phi_{-}^{x}(z)+\frac{1}{c}\sum_{x\in D}c_{-x}v_{x}^{t}z^{-x}.$$

Let $P_{D}$ denote the projection of Fourier coefficients of a typical *f*_−_(*z*) for |*z*| < *R*_−_ to the set D, then equation (3.19) leads to

3.20 $$\sum_{x\in D}\left(1-\frac{c_{-x}}{c}\right)v_{x}^{t}z^{-x}+\sum_{x\in D}\frac{c_{-x}}{c}v_{x}^{t}P_{D}\left(\frac{\phi_{-}^{x}}{L_{-}}\right)(z)=\frac{P_{D}(\frac{v_{-}^{i}}{L_{-}})(z)}{L_{+}(z_{P})},\;|z|<R_{-},$$

which yields an *N* × *N* system of linear algebraic equations for ${\left\{v_{x}^{t}\right\}}_{D}$, i.e. the unknowns ${\left\{v_{x}^{s}\right\}}_{D}$, since ${\left\{v_{x}^{i}\right\}}_{D}$ are known in terms of the incident wave (2.2). Indeed, with the notation $C_{\kappa }(p)$ to denote the coefficient of $z^{\kappa }$ for polynomials *p* of the form $C_{1}z+C_{2}z^{2}+\ldots ,$ we get

3.21 $$\begin{array}{rl} & \sum_{\nu =1}^{N}\left(1-\frac{c_{\nu }}{c}\right)\delta_{\kappa \nu }v_{-\nu }^{t}z^{\kappa }+\sum_{\kappa =1}^{N}\sum_{\nu =1}^{N}\frac{c_{\nu }}{c}v_{-\nu }^{t}C_{\kappa }\left(P_{D}\left(\frac{\phi_{-}^{-\nu }}{L_{-}}\right)\right)z^{\kappa }\\ & \;=L_{+}^{-1}(z_{P})\sum_{\kappa =1}^{N}C_{\kappa }\left(P_{D}\left(\frac{v_{-}^{i}}{L_{-}}\right)\right)z^{\kappa },\;|z|<R_{-}. \end{array}$$

The above equation can be written in a symbolic manner as

3.22 $$a_{\kappa \nu }\chi_{\nu }=b_{\kappa }\;\;(\kappa ,\nu =1,\ldots ,N),$$

where

3.23 $$\begin{array}{rl} & a_{\kappa \nu }=\left(1-\frac{c_{\nu }}{c}\right)\delta_{\kappa \nu }+\frac{c_{\nu }}{c}C_{\kappa }\left(P_{D}\left(\frac{\phi_{-}^{-\nu }}{L_{-}}\right)\right)\\ \;\text{and}\; & \chi_{\nu }=v_{-\nu }^{t},\;b_{\kappa }=L_{+}^{-1}(z_{P})C_{\kappa }\left(P_{D}\left(\frac{v_{-}^{i}}{L_{-}}\right)\right). \end{array}\}$$

Formally, applying the inversion of the coefficient matrix in (3.22), i.e. **χ** = **A**^−1^**b**, gives ${\left\{v_{x}^{t}\right\}}_{x\in D}$; substitution of this expression back in (3.18), via (3.6), as well as (3.14) leads to the complete solution of the Wiener–Hopf equation. Let ${\tilde{a}}_{\nu \kappa }$ denote the components of the inverse of **A**. Then

3.24 $$v_{x}^{t}={\tilde{a}}_{-x\kappa }L_{+}^{-1}(z_{P})C_{\kappa }\left(P_{D}(\frac{v_{-}^{i}}{L_{-}})\right).$$

The expression (3.24) has been verified using a numerical solution (based on the scheme described in appendix D of [12]) of the discrete Helmholtz equation (2.4) and assumed conditions on the crack faces for several choices of the damaged links; we omit the graphical plots of the comparison as they are indistinguishable on the considered graph scale.

**Remark 3.1.**

When *ω*_2_ (=ℑω) is positive, it follows from the Krein conditions that there exists a unique solution in square summable sequences since only a finite number *N* of damaged links are present. This is a statement on the lines of that provided by Sharma for the sharp crack tip [12,13] and the rigorous results of Ando *et al.* [75]. The limiting case as *N* → ∞ can be a different story altogether and it is not pursued here.

## 4. Examples of specific damage zones

Choosing different values of the coefficients *c*_−*j*_, *j* ∈ [1, *N*] one can consider various damage zones. Some of them are discussed below.

### (a) Completely destroyed zone

Consider the simplest case when *c*_−*x*_ ≡ 0. (In fact, it is a bad choice of the left-hand side of the cohesive zone.) Then (3.20) reduces to (using (3.9))

4.1 $$\sum_{x\in D}v_{x}^{t}z^{-x}=L_{+}^{-1}(z_{P})P_{D}\left(\frac{v_{-}^{i}}{L_{-}}\right)(z),\;|z|<R_{-},$$

but $\sum_{x\in D}v_{x}^{t}z^{-x}=P_{D}(v_{-}+v_{-}^{i})(z)$, so that it is a special case of the complete exact solution given in [12], i.e. $v_{-}(z)=(L_{+}^{-1}(z_{P})L_{-}^{-1}(z)-1)v_{-}^{i}(z)$, |*z*| < *R*_−_ (see equation (2.29) in [12] and equation (4.1b) in [13]). The detailed analysis and expressions of the solution based on the latter appear in [13], where the single crack was considered. It is natural, as this special case corresponds to a single (slightly longer) crack.

### (b) ‘Healthy’ (no damage) zone

For the case *c*_−*x*_ ≡ *c*, the above extra equation (3.20) arises again due to a ‘bad choice’ of the origin (cohesive crack tip) to define the half-Fourier transforms! Consider the simplest case when *c*_−*x*_ ≡ *c*. Evidently, this case coincides with the previous one when *c*_−*x*_ ≡ 0, except for a shift in the origin from (0, 0) to ( − *N*, 0) (a single slightly shorter crack). Then (3.20) reduces to (using (3.9))

4.2 $$\sum_{x\in D}v_{x}^{t}P_{D}L_{-}^{-1}\sum_{m=0}^{-x-1}{\bar{l}}_{+m}z^{-m}z^{-x}=L_{+}^{-1}(z_{P})P_{D}L_{-}^{-1}v_{-}^{i},\;|z|<R_{-}.$$

With the substitution $z\mapsto z^{-1},x\mapsto -x$ in the above equation, we get

4.3 $$\sum_{x=1}^{N}v_{-x}^{t}P_{D}L_{+}^{-1}(z)\sum_{m=0}^{x-1}{\bar{l}}_{+m}z^{m}z^{-x}=L_{+}^{-1}(z_{P})P_{D}L_{+}^{-1}(z)v_{-}^{i}(z^{-1}),\;|z|>R_{-}^{-1},$$

i.e.

4.4 $$\sum_{x=1}^{N}v_{-x}^{t}\sum_{m=0}^{x-1}{\bar{l}}_{+m}z^{m}z^{-x}=L_{+}^{-1}(z_{P})v_{-}^{i}(z^{-1}),\;|z|>R_{-}^{-1},$$

and, finally,

4.5 $$\sum_{x=1}^{N}v_{-x}^{t}z^{-x}L_{+}^{-1}(z)=L_{+}^{-1}(z_{P})v_{-}^{i}(z^{-1}),\;|z|>R_{-}^{-1}.$$

Here, the reference expression from [12,13] is $v_{+}=(1-L_{+}^{-1}(z_{P})L_{+})v_{-}^{i},|z|>R_{+}$, with which it agrees.

### (c) A zone with continuously distributed damage

Let us consider a relatively general case that models real damage accumulation in the damage zone. In this case, one can reasonably assume that at the crack tip the stiffness of the interfacial zone is minimal (the damage is most pronounced), then increases monotonically and, finally, at the other end of the zone, it takes the same magnitude as a non-damaged lattice. A typical representative of such an interface is the exponential distribution

$$c_{-x}=c\exp\left(\frac{\alpha x}{N}\right),\;x\in D.$$

**Figure 3.**
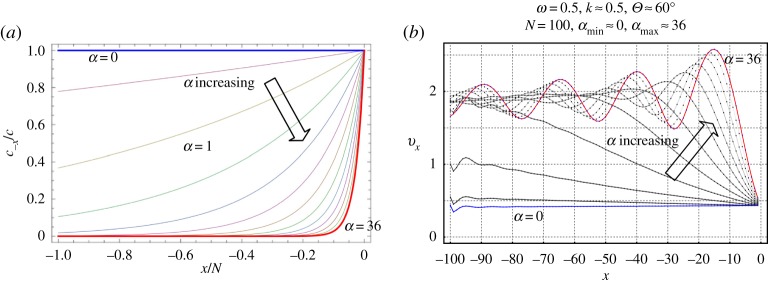
(*a*) Illustration of *c*_−*x*_ with $c_{-x}=c\exp(\alpha x/N),x\in D$. (*b*) Illustration of (total) *v*_*x*_ given by (3.17) for *N* = 100. The curves in blue and red correspond to the minimum and maximum values of *α*, respectively. (Online version in colour.)

**Figure 4.**
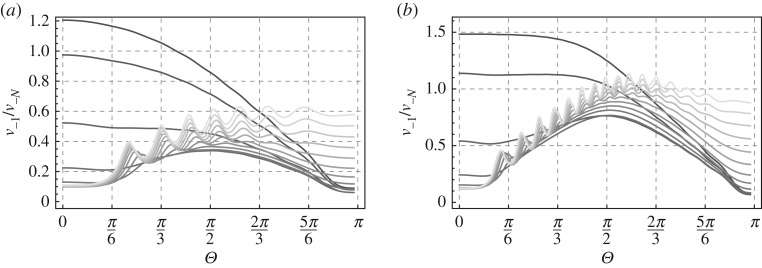
Ratio of the amplitudes at the ends of the damage zone of length *N* = 40. (*a*) corresponds to *ω* = 0.6. (*b*) represents the other normalized frequency *ω* = 1.2. Plots for a range of the parameter *α* = {10^−6^, 0.25, 1, 2.25, 4, 6.25, 9, 12.25, 16, 20.25, 25, 30.3, 36} with a darker shade for smaller *α*.

The parameter *α* regulates the rate of damage accumulation. Note that *α* ≫ 1 and *α* ≪ 1 correspond to part (a) and part (b) of this section, respectively. Figure 3 shows an illustration of $v_{x}^{t}$ given by (3.24) for *N* = 100. It is emphasized here that the graphical results for the same choice can be obtained using the numerical scheme (described in appendix D of [12]), and these are found to coincide with the plot in figure 3*b*. As one can see, the presence of a high gradient in the elastic properties of the cohesive zone significantly amplifies the local scattered field near the tip of the zone. As a result, pronounced damage should be expected exactly here that is consistent with the assumptions. However, when *α* is close enough to zero, the opposite phenomenon happens as now the gradient is small while the jump of the material properties undergoes its maximum value (in fact, it is equivalent to the second case above). It is thus important to compare which part of the damage zone can be subjected to higher risk for further damage. It is also evident that the angle of the incident wave *θ* may essentially influence the discussed effect. Respective graphical results for the ratio *v*_−1_/*v*_−*N*_ are presented in figure 4 and show the impact of the incident wave frequency by considering two different normalized values *ω* = 0.6 and *ω* = 1.2. As expected large and small values of the parameter *α* determining the damage gradient inside the zone change the effect significantly. Namely for small values of *α* the left-hand end of the damage zone is impacted by higher amplitudes and vice versa. At the right-hand end of the zone (contacting the undamaged part of the zone), the effect is less straightforward. Also for the incident waves parallel to the crack (*θ* = 0 and *θ* = *π*) the results are different. The first type can, in fact, be interpreted as the so-called feeding waves (e.g. [30]) for the dynamic case.

**Figure 5.**
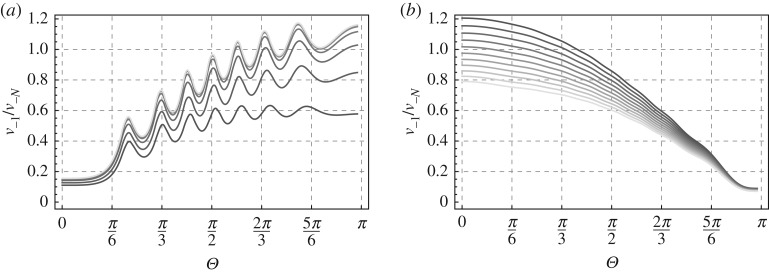
Ratio of the amplitudes at the ends of the damage zone of length *N* = 40 and frequency *ω* = 0.6. (*a*) corresponds to large values of *α* = {36, 64, 100, 144, 196, 256, 324, 400, 484, 576, 676}, while (*b*) corresponds to small values *α* = {10^−6^, 0.05, 0.1, 0.15, 0.2, 0.25, 0.3, 0.35, 0.4, 0.45, 0.5}. Plots for a range of *α* with a darker shade for smaller *α*.

In figure 5, we show in more detail the influence of the big and small values of the parameter *α*. Exact values of the parameters are depicted in the captions of the respective figures.

### (d) Damage represented by a bridge crack

**Figure 6.**
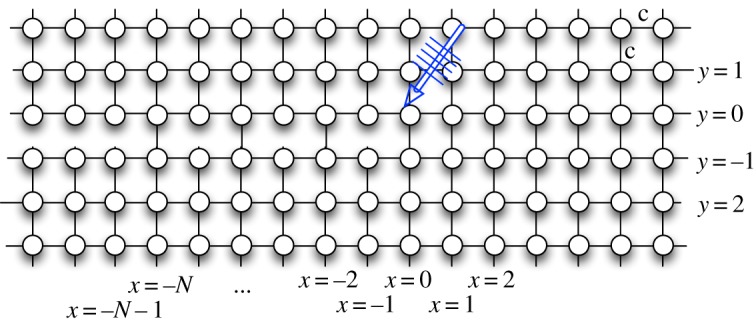
Geometry of the square lattice structure with a partially open crack tip and *N* = 2 and *M* = 6. (Online version in colour.)

Let *N* be even. In the following, we will use the standard notation:

4.6 $$\begin{array}{rl} & Z^{+}=\{0,1,2,\ldots \},\;Z^{-}=\{-1,-2,\ldots \},\\ & Z_{e}=\{0,\pm 2,\pm 4,\ldots \},\;Z_{o}=\{\pm 1,\pm 3,\ldots \}\\ \;\text{and}\; & Z_{S}^{-}=Z_{-}\setminus D,\;Z_{S}^{+}=Z_{+}\cup D, \end{array}\}$$

for different subsets of the set of entire numbers. Consider the case when

$$c_{-x}=c,\;x\in D\cap Z_{e}\;\text{and}\;c_{-x}=0,\;x\in D\cap Z_{o}$$

(figure 6). Here, $\max|D\cap Z_{e}|$ is *N*, which is replaced by 2*M* for convenience; thus the intact bonds on the even sites in the cracked row begin at *x* = −2*M*. The difference between (2.7) and (2.8) becomes

4.7 $$\begin{array}{rl} & c(2u_{x,1}^{s}+v_{x+1,0}^{s}+v_{x-1,0}^{s}+(-5+\omega^{2})v_{x,0}^{s})+2(c-cH(x+2M)\delta_{x,e}-cH(x)\delta_{x,o})v_{x}^{s}\\ & \;=-2(c-cH(x+2M)\delta_{x,e}-cH(x)\delta_{x,o})v_{x}^{i}. \end{array}$$

Using the Fourier transform (2.12) to equation (4.7) and taking into account the following representations of the functions $u^{F}(z)={\left(u_{x}^{s}\right)}^{F}$, $v^{F}(z)={\left(v_{x}^{s}\right)}^{F}$:

4.8 $$\begin{array}{rl} & u^{F}(z)\equiv u^{+}(z)+u^{-}(z),\;u^{\pm }(z)=\sum_{Z^{\pm }}z^{-x}u_{x},\;z\in A, \end{array}$$

4.9 $$\begin{array}{rl} & v^{F}(z)=v^{+}(z)+v^{-}(z),\;v^{\pm }(z)=z^{2M}v_{e}^{\pm }(z)+v_{o}^{\pm }(z), \end{array}$$

4.10 $$\begin{array}{rl} & v_{e}^{\pm }(z)=z^{-2M}\sum_{Z_{S}^{\pm }\cap Z_{e}}z^{-x}v_{x}=\sum_{Z^{\pm }\cap Z_{e}}z^{-x}v_{x},\;z\in A \end{array}$$

4.11 $$\begin{array}{rl} \;\text{and}\; & v_{o}^{\pm }(z)=\sum_{Z^{\pm }\cap Z_{o}}z^{-x}v_{x},\;z\in A, \end{array}$$

we get

4.12 $$d^{-1}(z)=\lambda (z)-1-Q(z)=-(\lambda^{-1}+1)$$

and

4.13 $$d{\left(z\right)}^{-1}(z^{2M}v_{\text{e}}^{+}+v_{o}^{+}+z^{2M}v_{\text{e}}^{-}+v_{o}^{-})+2(z^{2M}v_{\text{e}}^{-}+v_{o}^{-})=-2(z^{2M}v_{\text{e}}^{i-}+v_{o}^{i-}),$$

where we have taken into account that z↦−z, since $v_{\text{e}}^{+}(z)=v_{\text{e}}^{+}(-z)$ while $v_{o}^{+}(z)=-v_{o}^{+}(-z)$. Thus, we obtain the matrix Wiener–Hopf equation

4.14 $$\text{A}(z){\text{v}}^{+}(z)+\text{B}(z){\text{v}}^{-}(z)=\text{f}(z),\;z\in A,$$

where we have defined new plus and minus vector functions ${\text{v}}^{\pm }(z)={\left(v_{e}^{\pm },v_{o}^{\pm }\right)}^{⊤}$. The components of the matrices **A**(*s*), **B**(*s*) and the right-hand side of equation (4.14) are

4.15 $$\begin{array}{rl} & a_{11}=1,\;b_{11}=(1+2d(z)),\;a_{12}(z)=z^{-2M},\;\;b_{12}(z)=z^{-2M}(1+2d(z)),\\ & a_{21}=z^{2M},\;b_{21}=z^{2M}(1+2d(-z)),\;a_{22}(z)=-1,\;b_{22}(z)=-(1+2d(-z)) \end{array}$$

and

4.16 $$f_{1}(z)=-2(v_{\text{e}}^{i-}-z^{-2M}v_{o}^{i-})d(z),\;f_{2}(z)=-2(z^{2M}v_{\text{e}}^{i-}+v_{o}^{i-})d(-z),$$

where *d*(*z*) has already been defined in (4.12). Note **B** = **A** + **D**, *d*_11_ = 2*d*(*z*), *d*_12_ = *z*^−2*M*^2*d*(*z*), *d*_21_ = *z*^2*M*^2*d*( − *z*), *d*_22_ = −2*d*( − *z*). Let

4.17 $$\begin{array}{rl} \text{C} & =\text{I}+{\text{A}}^{-1}\text{D}=\text{I}+\frac{1}{\det\text{A}}\left(\begin{array}{cc} a_{22} & -a_{12}\\ -a_{21} & a_{11} \end{array}\right)\left(\begin{array}{cc} d_{11} & d_{12}\\ d_{21} & d_{22} \end{array}\right)\\ & =\text{I}-\left(\begin{array}{cc} d(z) & 0\\ 0 & -d(-z) \end{array}\right)\left(\begin{array}{cc} 1 & z^{-2M}\\ -z^{2M} & 1 \end{array}\right)=\left(\begin{array}{cc} 1-d(z) & -d(z)z^{-2M}\\ -d(-z)z^{2M} & 1+d(-z) \end{array}\right). \end{array}$$

Equation (4.14) can be rewritten in an equivalent form

4.18 $${\text{v}}^{+}(z)+\text{C}(z){\text{v}}^{-}(z)={\text{A}}^{-1}(z)\text{f}(z),\;z\in A,$$

where **C**(*z*) = **A**^−1^(*z*)**B**(*z*). The matrix **C** possesses a structure which in general does not admit factorization by standard techniques for arbitrary *N* (for *N* = 1, perhaps).

On the other hand, as has been proven above, this special case can be reduced to the solution of *N* linear algebraic equations (see also [56]). For example, the problem with a cohesive zone of similar geometry in *continuous formulation* [76] cannot be reduced to a scalar Wiener–Hopf problem and requires an application of other numerical techniques [57,71,72,77].

**Figure 7.**
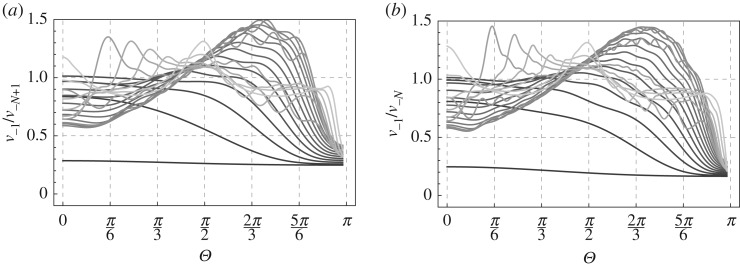
Length of the damage zone: *N* = 40. Bridged bonds are x=−1,−3,…,−N+1 and bonds intact: x=−2,−4,…,−N. Plots for a range of frequencies of the incident waves: *ω* = {0.01, 0.1, 0.2, 0.3, 0.4, 0.5, 0.6, 0.7, 0.8, 0.9, 1.0, 1.1, 1.2, 1.3, 1.4, 1.5, 1.6, 1.7, 1.8, 1.9, 1.95}, with a darker shade used for smaller *ω*.

In figure 7, we show the ratio of amplitudes in the last two points on the left-hand side of the damage zone to that on the right-hand side of the zone (*x* = 0). Exact values of the parameters are depicted in the captions of the respective figures. Now we examine in more detail the impact of the frequency of the incident waves.

In the context of the matrix kernel (4.17), with the distinguished presence of the off-diagonal factors *z*^−2*M*^ and *z*^2*M*^, the reduction to the linear algebraic equation obtained above is reminiscent of that proposed for the Wiener–Hopf kernel with exponential phase factors that appear in several continuum scattering problems in fluid mechanics and fracture mechanics [52–55], and their discrete analogues in the form of scattering due to a pair of staggered cracks and rigid constraints [34,56,78]; both of these are based on an exact solution of the corresponding staggerless case [35,79–81].

## 5. Reconstruction of the scattered field

Let C be a contour in the annulus A. By the inverse Fourier transform $u_{x,y}^{s}=(1/2\pi i)\int_{C}u_{y}^{F}(z)z^{x-1}\;\text{d}z$, i.e.

5.1 $$u_{x,y}^{s}=\frac{1}{2\pi i}\frac{1}{2}\int_{C}v^{F}(z)\lambda^{y}(z)z^{x-1}\;\text{d}z,\;x\in Z,\;y=0,1,2,\ldots ,$$

where *v*^*F*^ is given by (3.15). For $y=0,1,2,\ldots ,u_{x,-y-1}^{s}=-u_{x,y}^{s}$, x∈Z, due to skew symmetry. The total wave field is given by

5.2 $$u_{x,y}=u_{x,y}^{s}+u_{x,y}^{i},\;x\in Z,\;y\in Z.$$

Concerning the effect of the damage, using the decomposition $v^{F}(z)=v_{a}^{F}(z)+v_{P}^{F}(z)$ (3.15), it is easy to see that $v_{a}^{F}(z)$ coincides with the solution given in [12], i.e. it describes the scattering due to an undamaged crack tip; thus, the effect of the damage zone is represented by the second term $v_{P}^{F}(z)$ in (3.15).

The perturbation in the scattered field (5.1) induced by the damage zone is given by

5.3 $$\begin{array}{rl} {\hat{u}}_{x,y} & =\frac{1}{2\pi i}\frac{1}{2}\int_{C}v_{P}^{F}(z)\lambda^{y}(z)z^{x-1}\;\text{d}z,\;x\in Z,\;y=0,1,2,\ldots ,\\ & =\frac{1}{2\pi i}\frac{1}{2}\int_{C}(L_{+}C_{+}^{P}+L_{-}^{-1}C_{-}^{P})\lambda^{y}(z)z^{x-1}\;\text{d}z\\ & =\frac{1}{2\pi i}\frac{1}{2}\int_{C}\left(-\frac{1}{c}\sum_{m\in D}c_{-m}v_{m}^{t}(L_{+}\phi_{+}^{m}+L_{-}^{-1}\phi_{-}^{m})+\frac{1}{c}\sum_{m\in D}c_{-m}v_{m}^{t}z^{-m}\right)\lambda^{y}(z)z^{x-1}\;\text{d}z\\ & =\frac{1}{2L_{+}(z_{P})}\sum_{m\in D}\frac{c_{-m}}{c}{\tilde{a}}_{-m\kappa }C_{\kappa }\left(P_{D}\frac{v_{-}^{i}}{L_{-}}\right)\frac{1}{2\pi i}\int_{C}\Lambda_{m}(z)\lambda^{y}(z)z^{x-1}\;\text{d}z, \end{array}$$

where

5.4 $$\Lambda_{m}(z)=z^{-m}-(L_{+}(z)\phi_{+}^{m}(z)+L_{-}^{-1}(z)\phi_{-}^{m}(z)),\;m\in D.$$

For $\xi \sqrt{x^{2}+y^{2}}\gg 1$ and *ω*/*c* ∈ (0, 2), where *ξ* ∼ *ω*/*c* is related to the wavenumber of an incident wave, a far-field approximation of the exact solution (5.1) can be constructed; also an analogous result holds for $\omega /c\in (2,2\sqrt{2})$. It is sufficient for our purposes to focus on the effect of the damage zone D so that we investigate the far-field approximation of (5.3), i.e. mainly associated with the expression of Λ_*m*_ given by (5.4) for each m∈D. Following [12], the approximation of the far field can be obtained using the stationary phase method [82]. The substitution *z* = e^−*iξ*^ maps the contour C into a contour *C*_*ξ*_. In terms of polar coordinates (*R*, *θ*), the lattice point (*x*, *y*) can be expressed as

5.5 x=Rcos⁡θ,y=Rsin⁡θ.

Let

5.6 $$\Phi (\xi )=\eta (\xi )\sin\theta -\xi \cos\theta ,\eta (\xi )=-i\log\lambda ({\text{e}}^{-i\xi }).$$

The function Φ (5.6) possesses a saddle point [83,84] at *ξ* = *ξ*_*S*_ on *C*_*ξ*_, with Φ′(*ξ*_*S*_) = *η*′(*ξ*_*S*_)sin*θ* − cos*θ* = 0, Φ″(*ξ*_*S*_) = *η*″(*ξ*_*S*_)sin*θ* ≠ 0, which is the same as that discussed in [12]. Omitting the details of the calculations, it is found that

5.7 $${\hat{u}}_{x,y}∼\frac{1}{2\sqrt{\pi }}\frac{1+i\text{ sign}(\eta^{\prime \prime }(\xi_{S}))}{2c}\frac{\lambda^{y}({\text{e}}^{-i\xi_{S}}){\text{e}}^{-i\xi_{S}(x-1)}}{{\left(R|\eta^{\prime \prime }(\xi_{S})|\sin\theta \right)}^{1/2}}\sum_{m\in D}(c_{-m}v_{m}^{t}\Lambda_{m}({\text{e}}^{-i\xi_{S}})),$$

as *ωR*/*c* → ∞. The expression (5.7) has been verified using a numerical solution of the discrete Helmholtz equation (based on the scheme described in appendix D of [12]); a graphical demonstration of the same is omitted in the paper.

## 6. Concluding remarks

We have shown how the scattering problem in a square lattice with an infinite crack with a damage zone near the crack tip of arbitrary properties can be effectively solved by We were able to reduce it to a scalar Wiener–Hopf method. We have applied a new method that uses specific discrete properties of the system under consideration. It consists of solving an auxiliary *N* × *N* system of linear equations with a unique solution (remark 3.1). The effectiveness of the method has been highlighted by some numerical examples and the constructed asymptotic expression of the scattered field at infinity. Analysis of the solution near two ends of the damage zone and at infinity can be used in a non-destructive testing procedure, among other applications. The method may be useful for solving other matrix Wiener–Hopf problems appearing in the analysis of the dynamics of discrete structures with defects. Indeed, the discrete scattering problem for the bridge damage zone has been written in a vectorial problem with a 2 × 2 matrix-kernel and has simultaneously transformed it, by the aforementioned approach, to a scalar one (modulo the accompanying linear algebraic equation). This gives rise for a hope for building a closed form standard procedure that allows for effective factorization of similar matrices of an arbitrary size.

## Data accessibility

There are no data in the paper. All the plots are based on expressions provided and are not dependent on a numerical code/software.
